# Regulation of Ebola virus VP40 matrix protein by SUMO

**DOI:** 10.1038/srep37258

**Published:** 2016-11-16

**Authors:** Maite Baz-Martínez, Ahmed El Motiam, Paula Ruibal, Gabriela N. Condezo, Carlos F. de la Cruz-Herrera, Valerie Lang, Manuel Collado, Carmen San Martín, Manuel S. Rodríguez, Cesar Muñoz-Fontela, Carmen Rivas

**Affiliations:** 1 Centro de Investigación en Medicina Molecular (CIMUS), Universidade de Santiago de Compostela, Instituto de Investigaciones Sanitarias (IDIS), Santiago de Compostela, E15706, Spain; 2 Heinrich Pette Institute, Leibniz Institute for Experimental Virology, Martinistraβe 52, D20251, Hamburg, Germany; 3 Bernhard Nocht Institute for Tropical Medicine, Bernhard-Nocht Str 74, D20359, Hamburg, Germany; 4 Department of Macromolecular Structures and NanoBioMedicine Initiative, Centro Nacional de Biotecnología-CSIC, Darwin 3, Madrid 28049, Spain; 5 Department of Molecular and Cellular Biology, Centro Nacional de Biotecnología-CSIC, Darwin 3, Madrid 28049, Spain; 6 Ubiquitylation and Cancer Molecular Biology laboratory, Inbiomed, San Sebastian-Donostia, 20009 Gipuzkoa, Spain; 7 Instituto de Investigación Sanitaria de Santiago de Compostela (IDIS), Complexo Hospitalario Universitario de Santiago de Compostela (CHUS), SERGAS, Santiago de Compostela, E15706, Spain; 8 Advanced Technology Institute in Life Sciences (ITAV) CNRS-USR3505, 31106 Toulouse, France; 9 University of Toulouse III-Paul Sabatier, 31077, Toulouse, France

## Abstract

The matrix protein of Ebola virus (EBOV) VP40 regulates viral budding, nucleocapsid recruitment, virus structure and stability, viral genome replication and transcription, and has an intrinsic ability to form virus-like particles. The elucidation of the regulation of VP40 functions is essential to identify mechanisms to inhibit viral replication and spread. Post-translational modifications of proteins with ubiquitin-like family members are common mechanisms for the regulation of host and virus multifunctional proteins. Thus far, no SUMOylation of VP40 has been described. Here we demonstrate that VP40 is modified by SUMO and that SUMO is included into the viral like particles (VLPs). We demonstrate that lysine residue 326 in VP40 is involved in SUMOylation, and by analyzing a mutant in this residue we show that SUMO conjugation regulates the stability of VP40 and the incorporation of SUMO into the VLPs. Our study indicates for the first time, to the best of our knowledge, that EBOV hijacks the cellular SUMOylation system in order to modify its own proteins. Modulation of the VP40-SUMO interaction may represent a novel target for the therapy of Ebola virus infection.

Ebola virus (EBOV), the causing agent of Ebola virus disease (EVD) epidemics in West Africa, causes severe febrile disease in humans and non-human primates, resulting in high case-fatality ratios^1^,^2^. EBOV particles consist of seven structural proteins, including the matrix protein VP40. This is the most abundant protein in virions and expression of the protein alone in mammalian cells induces production of particles with a density similar to that of virions^3^,^4^,^5^. VP40 has been shown to be important for EBOV budding as well as for virus structure and stability^4^,^5^. In addition, VP40 also regulates viral genome replication and transcription and it has been proposed to influence cellular gene expression^6^. VP40 is made up of 326 amino acids and consists of two domains connected by a flexible linker. The N-terminal domain is responsible for oligomerization of VP40, while the C-terminal domain is required for membrane binding^7^,^8^. Oligomerization of VP40 has also been shown to be required for efficient membrane binding by VP40, as well as its transport to the surface and subsequent particle formation^9^. In order to oligomerize, VP40 has to undergo two major conformational changes: movement of the C-terminal domain and displacement of residues 31 to 70 of the N-terminal region^10^.

Post-translational modifications such as the conjugation of ubiquitin-like proteins can induce conformational changes of the target proteins enhancing its functional repertoire^11^. This is probably one of the reasons why viruses use the cellular SUMOylation and ubiquitination pathways to regulate their own proteins. In the case of Ebola infection, EBOV VP35 protein induces the SUMOylation of IRF7 to disrupt antiviral responses^12^. In addition, ubiquitin itself is thought to be exploited by EBOV to facilitate efficient virus egress^3^,^13^,^14^. However, usurpation of the SUMOylation system by EBOV to regulate its own proteins has not been reported so far.

SUMO is a member of the family of ubiquitin-like proteins, which shares about 18% sequence identity to ubiquitin, and is structurally quite similar^15^. Analogous to ubiquitination, post-translational modifications with SUMO proteins involve isopeptide bond formation between the carboxyl group of the modifier and the ε-amino group of a lysine residue in the target. Usually the target lysine for SUMO is located in the consensus sequence ψKxE (where ψ is a hydrophobic residue, and x any residue)^16^,^17^,^18^. However, SUMO can be also conjugated to lysine residues located in non-consensus sequences. To date, four SUMO isoforms (SUMO1 to SUMO4) have been discovered in mammals. In mammalian cells, SUMO1 is expressed at lower levels than SUMO2. SUMO2 and SUMO3 are very similar between them and can be conjugated to target proteins in a chain-wise fashion due to internal SUMO conjugation motifs, whereas SUMO1 lacks this ability^19^,^20^. SUMOylation regulates a wide range of processes such as protein stability or nucleus-cytoplasm transport but its main function is to regulate protein-protein interaction. Viral proteins were among the first substrates shown to be modified by SUMO and SUMOylation seems to facilitate viral infection in cells^21^,^22^.

Here we investigated whether SUMO interacts with EBOV VP40 and whether this interaction regulates its functional properties. We found that EBOV VP40 covalently interacts with SUMO *in vitro* and in transfected cells. We demonstrated that the lysine residue 326 in VP40 is involved in this interaction. Importantly, we found that mutation of this lysine residue in VP40 reduced its stability and abolished the incorporation of SUMO into the VP40-VLPs. In summary, our findings provide evidence of a new mechanism for VP40 regulation and suggest that the SUMO pathway is critical for the EBOV life cycle. Modulation of the SUMO-VP40 interaction may represent a novel target for therapeutics to block EBOV infection.

## Results

### Modulation of VP40 by SUMO

**Figure 1:**
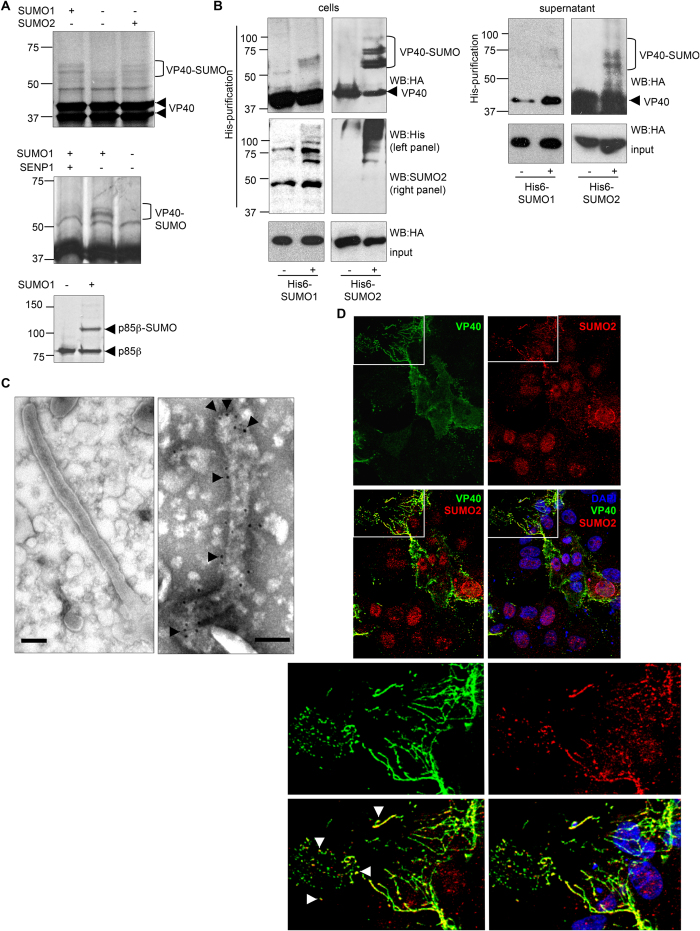
Covalent modification of EBOV VP40 by SUMO1 and SUMO2 *in vitro* and in transfected cells. (**A**) ^35^S-methionine labeled *in vitro* translated VP40 protein was used as a substrate in an *in vitro* SUMOylation assay in the presence of SUMO1 or SUMO2 (upper panel). De-conjugation of SUMO1 from VP40 by SENP1 (middle panel). *In vitro* SUMOylation assay of ^35^S-methionine labeled *in vitro* translated p85β protein carried out in similar conditions is shown as a positive control (lower panel). (**B**) HEK-293 cells were co-transfected with HA-VP40 and an empty vector, with HA-VP40, Ubc9 and His6-SUMO1 or with HA-VP40, Ubc9 and His6-SUMO2, as indicated. At 24 h after transfection total cellular protein extracts and Histidine purified cellular proteins were analyzed by Western-blotting using anti-HA, anti-Histidine (His) or anti-SUMO2 antibody, as indicated (left panel). Total cell culture supernatants and Histidine tagged proteins purified from cell culture supernatants were analyzed by Western-blotting using an anti-HA antibody (right panel). (**C**) HEK-293 cells were transfected with HA-VP40 and 72 h after transfection VLPs were purified as described in Materials and Methods. Purified VLPs were treated with Triton (right panel) or left untreated (left panel) and immunostained with rabbit anti-SUMO2 antibody and a secondary anti-rabbit antibody conjugated with colloidal gold (arrows). The bar represents 100 nm. D, Vero cells were transfected with HA-VP40. At 24 h after transfection cells were fixed and stained with anti-HA and anti-SUMO2 antibodies, and DAPI. Lower panels are higher-magnification images of the areas comprised by the white rectangle in the upper panels. SUMO2 was co-localized with VP40 in the cell projections (white arrowheads).

To analyze the putative modification of VP40 by SUMO we performed *in vitro* SUMOylation assays using *in vitro* translated [^35^S]methionine-labeled VP40 protein as a substrate. In the absence of SUMO, we detected unmodified VP40 protein migrating as a double band at around 40 kDa, as expected^3^,^4^. When the reaction was incubated with SUMO1 or SUMO2 we observed a higher molecular weight double band of around 60 kDa (Fig. 1A upper panel), which disappeared after incubation of the reaction with the recombinant SUMO specific protease SENP1 (Fig. 1A, middle panel). These results demonstrated that VP40 is SUMOylated *in vitro* by SUMO1 and SUMO2. In addition, the presence of a sole double VP40-SUMO1 band in the *in vitro* assay suggested that SUMOylation sites are limited and occurs at one lysine residue at a time. Note that the VP40-SUMO bands detected in the *in vitro* SUMOylation reaction were very faint in comparison with the SUMOylated bands detected when a positive control (p85β) was assayed under the same conditions (Fig. 1A lower panel). These results indicated that VP40 is a poor substrate for *in vitro* SUMOylation and suggested that an additional cell factor may be critical for VP40 SUMOylation. Then, in order to determine whether VP40 is conjugated to SUMO in cells, HEK-293 cells were co-transfected with HA-VP40 together with the SUMO-conjugating enzyme Ubc9 and His6-SUMO1, His6-SUMO2, or control empty pcDNA plasmids. At 24 h after transfection, His6-tagged proteins were purified in denaturing conditions using nickel beads. Western-blot analysis of the purified extracts with anti-HA antibody revealed bands of the expected size corresponding to VP40-SUMO1 and VP40-SUMO2 only when cells were co-transfected with His6-SUMO1 or His6-SUMO2, respectively, indicating that VP40 is modified by SUMO1 and SUMO2 in transfected cells (Fig. 1B, left panel). The levels of SUMO2 modified protein were consistently higher than those modified by SUMO1, suggesting that VP40 is more susceptible to be modified by SUMO2 than by SUMO1 in cells. VP40 can mediate its own release from mammalian cells and has an intrinsic ability to form virus-like particles^3^. Therefore, we decided to evaluate whether SUMO2 molecules were released together with VP40 from mammalian cells. Interestingly, analysis of the His-tagged proteins present in the cell-free culture supernatants revealed the presence of bands corresponding to VP40 protein conjugated to SUMO2 (Fig. 1B, right panel). A band corresponding to VP40-SUMO1 was barely detected in the supernatant recovered from cells co-transfected with His6-SUMO1 and VP40, which is in agreement with the lower levels of VP40-SUMO1 protein detected in transfected cells (Fig. 1B, right panel). These results indicated that SUMOylated VP40 was released from the cells. To confirm the presence of SUMO2 into the VLPs, we carried out immunoelectron microscopy analysis in purified VP40 VLPs using anti-SUMO2 antibody. Only filamentous particles treated with Triton X-100 were labeled with anti-SUMO2 antibody (Fig. 1C), demonstrating that SUMO2 was incorporated into VP40-VLPs. Finally, we analyzed whether the endogenous SUMO2 protein was co-localizing with VP40 by immunofluorescence staining. SUMO2 was mainly detected in the nucleus of cells transfected or not with HA-VP40 (Fig. 1D). In addition, SUMO2 was also detected co-localizing with VP40 in the cell projections, regions of the cell where virus-like particles (VLPs) are assembled and released (Fig. 1D), supporting the data indicating that SUMO2 is incorporated into the VP40-VLPs.

### Lysine 326 in VP40 is implicated in the conjugation to SUMO

**Figure 2:**
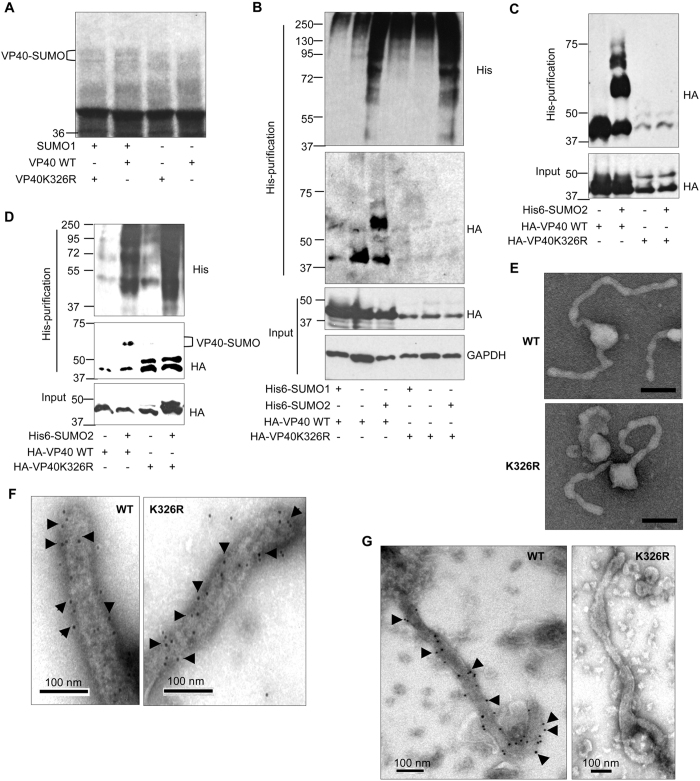
Lysine 326 in VP40 is implicated in SUMO conjugation. (**A**) ^35^S-methionine labeled *in vitro* translated VP40WT or VP40K326R protein were used as substrates in an *in vitro* SUMOylation assay in the presence of SUMO1. (**B**) HEK-293 cells were co-transfected with 0.4 μg of HA-VP40WT or HA-VP40K326R and an empty vector or Ubc9 and His6-SUMO1 or Ubc9 and His6-SUMO2. At 24 h after transfection total protein extracts and Histidine purified proteins were analyzed by Western-blotting using anti-HA or anti-Histidine (His) antibody, as indicated. (**C**) HEK-293 cells were co-transfected with HA-VP40WT or HA-VP40K326R and an empty vector or Ubc9 and His6-SUMO2. At 24 h after transfection cells were treated with MG132 for 4 hours. Total protein extracts and Histidine purified proteins were analyzed by Western-blotting using anti-HA antibody. (**D**) HEK-293 cells were co-transfected with HA-VP40WT (0.02 μg) or HA-VP40K326R (0.4 μg) and an empty vector or Ubc9 and His6-SUMO1 or Ubc9 and His6-SUMO2. At 24 h after transfection cells were treated with MG132 for 4 hours. Total protein extracts and Histidine purified proteins were analyzed by Western-blotting using anti-HA or anti-Histidine (His) antibody, as indicated. (**E**) HEK-293 cells were transfected with HA-VP40WT or HA-VP40K326R. At 72 h after transfection VLPs were purified as described in Materials and Methods. Negative staining electron microscopy images showing WT and K326R VLPs. The bar represents 100 nm. Purified VLPs were immunostained with anti-HA (**F**) or anti-SUMO2 (**G**) antibody and a secondary antibody conjugated with colloidal gold (arrows).

*In silico* analysis of VP40 using SUMOsp prediction tool pointed only to lysine 326 as the amino acid residue in VP40 from EBOV (*Zaire ebolavirus*) susceptible to conjugate to SUMO. Interestingly, a similar analysis carried out on VP40 from other ebolavirus species pointed to the same lysine residue as susceptible to conjugate to SUMO in *Sudan ebolavirus* (strain Gulu and Boniface), *Bundibugyo ebolavirus*, and *Tai Forest ebolavirus* but not in the presumably non-pathogenic for humans *Reston ebolavirus*. To test the involvement of this aminoacid on the SUMOylation of VP40 we generated a mutant in this lysine residue (VP40K326R) and evaluated its conjugation to SUMO. *In vitro* SUMOylation assays using *in vitro* translated [^35^S]methionine-labeled VP40WT or VP40K326R protein as substrates revealed the appearance of a faint double band of around 60 kDa corresponding with VP40WT-SUMO1, as above described (Fig. 2A). These bands were even fainter in the lane corresponding to the VP40K326R mutant (Fig. 2A), suggesting that K326 may be involved in the conjugation to SUMO. To evaluate this hypothesis we analyzed the SUMOylation of the WT and mutant proteins in transfected cells. Western-blot analysis of the His6-tagged proteins purified from HEK-293 cells transfected with HA-VP40WT, Ubc9, and His6-SUMO1 or His6-SUMO2 revealed the appearance of a faint band corresponding to VP40-SUMO1 protein and stronger VP40-SUMO2 bands, as we previously observed (Fig. 2B). We did not detect any VP40-SUMO band in cells transfected with the VP40K326R mutant, suggesting that the lysine residue 326 is the major amino acid residue implicated in its SUMO conjugation. Interestingly, we observed that the levels of the VP40K326R mutant were consistently lower than the levels of the WT protein, suggesting that mutation of the lysine residue 326 in VP40 reduced its stability (Fig. 2B). Therefore, we decided to evaluate the SUMOylation of VP40WT or VP40K326R in cells treated with the proteasome inhibitor MG132. Western-blot analysis of the His6-tagged proteins purified from HEK-293 cells transfected with HA-VP40WT, Ubc9, and His6-SUMO2 and incubated with MG132 revealed the appearance of strong VP40-SUMO2 bands, as expected (Fig. 2C). We did not detect SUMOylation of the VP40K326R mutant suggesting that the lysine residue 326 is involved in the conjugation of VP40 to SUMO (Fig. 2C). However, since we still detected lower levels of the mutant protein after MG132 treatment we decided to repeat the experiments transfecting lower amounts of HA-VP40WT plasmid (20 times less than the HA-VP40K326R plasmid) to force similar initial amounts of both, WT and mutant proteins in the SUMOylation assay. In these conditions, we still detect the SUMOylation of WT protein but not of the mutant one, confirming the involvemente of the lysine residue K326 in SUMO conjugation (Fig. 2D). We then analyzed the purified VP40WT or VP40K326R VLPs by immunoelectron microscopy with anti-HA and anti-SUMO2 antibody. We did not observe morphological differences between VP40 WT and VP40K326R VLPs after analysis by electron microscopy indicating that lysine residue K326 was not required for VP40 VLPs formation (Fig. 2E). In addition, we observed similar immunostaining of the VP40 WT and VP40K326R VLPs using anti-HA antibody (Fig. 2F). However, whereas SUMO2 was clearly detected in the VP40 WT VLPs we did not observe SUMO2 in the VP40K326R VLPs (Fig. 2G), suggesting the involvement of the lysine residue K326 in the recruitment of SUMO2 inside the VLPs.

### SUMOylation provides stability to VP40

**Figure 3:**
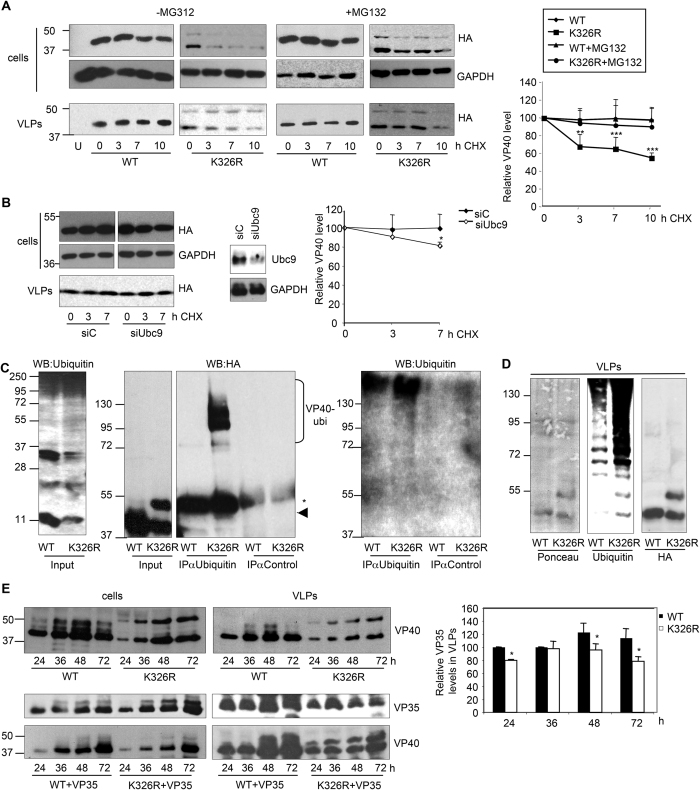
SUMOylation provides stability to VP40. (**A**) HEK-293 cells were transfected with HA-VP40WT or HA-VP40K326R and 24 after transfection cells were treated with cycloheximide (CHX) in the presence or absence of MG132. At the indicated time after treatment cells and supernatants containing VLPs were analyzed by Western-blotting using anti-HA antibody. Right panel, densitometric quantification of the VP40 protein in cells. Data are mean values +/− SE from at least three different experiments. **p < 0.005, ***p < 0.0005, Student’s t test. (**B**) HEK-293 cells were co-transfected with siC or siUbc9 and HA-VP40 WT and 48 after transfection cells were treated with cycloheximide (CHX). At the indicated times after treatment cells and supernatants containing VLPs were analyzed by Western-blotting using anti-HA antibody. Right panel, densitometric quantification of the VP40 protein in cells. Data are mean values +/− SE from at least three different experiments. *p < 0.05, Student’s t test. (**C**) Protein extracts from cells transfected with HA-VP40WT or HA-VP40K326R were subjected to immunoprecipitation (IP) with either mouse preimmune (control) serum or anti-ubiquitin antibody as indicated, total ubiquitinated proteins and VP40 were detected in the precipitated samples by Western-blot analysis using anti-Ubiquitin and anti-HA antibody, respectively. Controls for expression of VP40 and levels of endogenous ubiquitin are shown (input). The arrow or bracket indicates the ubiquitin conjugated VP40 protein. The star indicates the IgG band. (**D**) HEK-293 cells were transfected with HA-VP40WT or HA-VP40K326R. At 72 h after transfection VLPs were purified as described in Materials and Methods and analyzed by Western-blot using anti-ubiquitin or anti-HA antibody. (**E**) HEK-293 cells were transfected with HA-VP40WT or HA-VP40K326R in the presence or absence of EBOV HA-VP35. At different times after transfection cells and supernatants containing VLPs were analyzed by Western-blotting using anti-HA antibody. Right panel, densitometric quantification of the levels of VP35 detected in the VLPs relative to the levels detected at 24 h after transfection in the cells co-transfected with VP40WT, arbitrarily set at 100. Data are mean values +/− SE from at least three different experiments. *p < 0.05, Student's t test.

In order to examine the hypothesis of the VP40K326R reduced stability, HEK-293 cells were transfected with plasmids encoding HA-VP40WT or HA-VP40K326R, incubated with or without the proteasome inhibitor MG132, and treated with the protein synthesis inhibitor cycloheximide (CHX). Levels of VP40 in both, cell extracts and supernatants were analyzed at different times after treatment. WT protein was found to be relatively stable over the evaluated period in cells, independently of the MG132 treatment (Fig. 3A). In contrast, the levels of the mutant protein were significantly reduced at 3 h after treatment with CHX in the absence of MG132, and relatively stable in cells treated with MG132 (Fig. 3A). The levels of VP40WT or VP40K326R detected in the supernatant were similar at the different times after CHX treatment independently of the MG132 treatment (Fig. 3A). All together, these results indicated that the SUMOylation motif is involved in providing stability to VP40 and that the VP40K326R protein was being at least partially degraded by the proteasome. In addition, we also analyzed the levels of VP40 in both, cell extracts and supernatants after co-transfection with an siRNA targeting Ubc9 (siUbc9) or a control siRNA (siC) at different times after cyclohexamide treatment. Even though we did not detect any difference in the levels of VP40 in the supernatant of cells transfected with siUbc9 or the siC, the levels of the protein were significantly reduced in the cell extracts 6 h after CHX treatment when Ubc9 was knocked down (Fig. 3B), indicating that SUMO plays a role in the stability of VP40.

Interestingly, Western-blot analysis of the cell extracts transfected with VP40K326R revealed the appearance of a higher molecular weight form of around 50 kDa that appears to be more unstable than a lower form of 40 kDa and that remains during treatment with the proteasome inhibitor MG132 (Fig. 3A; see also Fig. 2B,C), suggesting that VP40K326R may be ubiquitinated. Remarkably, we also detected the 50 kDa mutant protein band in the cell supernatant suggesting that ubiquitinated VP40 protein is released to the cell supernatant.

To study the ubiquitination status of the SUMOylation mutant, we transfected HEK-293 cells with HA-VP40WT or HA-VP40K326R, and 24 h after transfection protein extracts were obtained and incubated with anti-ubiquitin antibody. Western-blot analysis with anti-HA antibody of the immunoprecipitated protein revealed several bands between 50–120 kDa in the lane corresponding to VP40K326R protein (Fig. 3C). However, we only detected a very faint band of around 80 kDa in the lane corresponding to VP40WT protein (Fig. 3C). These results indicated that the SUMOylation mutant protein was being highly ubiquitinated in comparison with the WT protein. In addition, the purified VP40WT and VP40K326R VLPs were evaluated by Western-blot analysis using anti-ubiquitin antibody. A clear increase in the levels of ubiquitinated proteins was observed in the VP40K326R VLPs in comparison with those detected in the VP40WT VLPs (Fig. 3D). In addition, the anti-ubiquitin antibody recognizes a band of around 50 kDa exclusively detected in the lane corresponding to the VP40K326R VLPs, suggesting that it may correspond to VP40K326R ubiquitinated protein (Fig. 3D). All together, these results indicated that mutation of the SUMOylation site in VP40 favors the interaction of VP40 with ubiquitin.

We speculated that the lower stability of the mutant VP40K326R may result in a reduced VLPs production. To test this hypothesis we decided to compare the recruitment of EBOV VP35 into the VP40 or VP40K326R derived VLPs. HEK-293T cells were co-transfected with VP40 WT or VP40K326R together with the empty vector pcDNA, or the plasmid encoding for EBOV VP35 (fused to HA, HA-VP35). At different times after transfection, cell extracts and cell-free culture supernatants containing released VLPs were harvested and proteins were analyzed by Western-blot. As expected, the levels of VP40K326R detected in cells were lower than the levels of the WT protein independently of the co-transfection of VP35 (Fig. 3E). Similarly, the levels of the VP40K326R protein detected in the cell supernatant were lower than the levels of VP40 WT (Fig. 3A). We did not observe differences in the expression of VP35 in cells transfected with VP40 WT or VP40K326R. However, the levels of VP35 found in the supernatant of cells transfected with VP40K326R were clearly reduced relative to the protein detected in the supernatant of VP40 WT transfected cells (Fig. 3E), indicating that mutation of the lysine K326R affects VLPs production.

## Discussion

Here, we investigated the regulation of the matrix protein VP40 of EBOV by SUMO proteins. We demonstrate that the VP40 protein is modified by SUMO1 and SUMO2 *in vitro* and in transfected cells, and that SUMO2 is incorporated into the VLPs, suggesting that SUMO has a relevant role in VP40 functions. *In silico* analysis of the putative SUMOylation sites in VP40 from the different *Ebolavirus* species pointed to a lysine residue located at the C terminal end of the protein as susceptible to be conjugated to SUMO. This lysine residue was not recognized as a putative SUMOylation site in the presumably non-pathogenic Reston virus, suggesting that the SUMOylation of VP40 may contribute to EBOV pathogenicity. The analysis of a mutant in this amino acid residue demonstrated that the lysine 326 was involved in VP40 stability as well as in the incorporation of SUMO2 into the VP40 VLPs. Although we cannot discard the involvement of other lysine residues in SUMO conjugation, our results indicate that indeed K326 has a role in VP40 SUMOylation.

The proteasome-mediated degradation of the VP40 SUMOylation mutant led us to evaluate its ubiquitination. Our results indicated that VP40 WT expressed in transfected cells was virtually not conjugated to ubiquitin, whereas the SUMOylation mutant exhibited ubiquitinated products. Furthermore, we detected ubiquitinated proteins in the purified VLPs that were more abundant when these VLPs contained the VP40 SUMOylation mutant. This result reinforces the idea that mutation of the SUMOylation site in VP40 increases its interaction with ubiquitin. It would be interesting to further determine the interplay between the different post-translational modifications of VP40 and to investigate whether SUMO may affect the interaction of VP40 with factors regulating these modifications^3^,^13^,^14^.

In summary, the results presented here extend our knowledge of the regulation of Ebola virus VP40 protein, pointing to the SUMOylation machinery of the cell as a relevant player and as a potential new therapeutic target for Ebola virus infection.

## Methods

### Cells, plasmids, transfections, and reagents

HEK-293, and Vero cells were cultured in complete medium (DMEM supplemented with 10% fetal bovine serum, 1% penicillin/streptomycin). The cells were transiently transfected using PEI (Polysciences, Inc) or lipofectamine (Invitrogen) transfection reagents, as suggested by the manufacturer. His6-SUMO1, His6-SUMO2, and SV5-Ubc9 plasmids were previously described^23^,^24^. EBOV VP40 was amplified and cloned into the pCMV5-HA vector using the oligonucleotides HA-VP40-WTF-5 GGGAATTCAGGCGGGTTATATTGCC-3 and HA-VP40WTR-5 GGGGATCCTTACTTCTCAATCACAGC-3. The resulting construct was designed HA-VP40. Oligonucleotides used to generate the pCMV5-HA-VP40K326R mutant were HA-VP40-WTF and HA-VP40K326R-5′- GGGGATCCTTACCTCTCAATCACAGC-3. DNA sequence of the inserts was confirmed by sequencing. Smart-pool siRNAs against Ubc9 (siUbc9) and scramble siRNA (siC) were purchased from Dharmacon.

### Antibodies

Anti-HA monoclonal and polyclonal antibodies were purchased from Covance and GenScript, respectively. Anti-SUMO2 was from Life Technologies, anti-ubiquitin was from Santa Cruz Biotechnology, and anti-GAPDH antibody was from Millipore.

### *In vitro* SUMO conjugation assay

*In vitro* SUMO conjugation assays were performed on [^35^S]methionine-labeled *in vitro*-transcribed/translated proteins as described previously^25^. Briefly, [^35^S]methionine-labelled proteins were incubated with E1 in a 10 μL reaction including an ATP regenerating system (50 mM Tris pH 7.6, 5 mM MgCl_2_, 2 mM ATP, 10 mM creatine phosphate, 3.5 U/mL of creatine kinase and 0.6 U/mL of inorganic pyrophosphatase), 10 μg SUMO1 or SUMO2, and 600 ng Ubc9. Reactions were incubated at 30 °C for 45 min. After terminating the reactions with SDS sample buffer containing β-mercaptoethanol, reaction products were fractionated by SDS-PAGE and detected by fluorography. The *in vitro* transcription/translation of proteins was performed by using 1 μg of plasmid DNA and a rabbit reticulocyte-coupled transcription/translation system according to the instructions provided by the manufacturer (Promega).

### *In vitro* deSUMOylation assay

*In vitro* deSUMOylation assay with recombinant GST-SENP1 was performed on VP40-SUMO1 or VP40-SUMO2 as described previously^26^. Briefly, SUMOylated proteins were incubated with 2 μg of GST-SENP1 (Biomol) in 30 μl reaction buffer containing 50 mM Tris (pH 7.5), 2 mM MgCl_2_ and 5 mM β-mercaptoethanol. Reactions were incubated at 37 °C for 1 h and terminated with SDS sample buffer containing mercaptoethanol. Reactions products were then fractionated on an 10% SDS-polyacrylamide gel, dried for 1 h, and exposed to X-ray film.

### Western blot analysis

Cells were washed in phosphate-buffered saline (PBS), scraped into SDS-PAGE loading buffer and boiled for 5 min. Proteins of total extracts were separated by SDS-PAGE and transferred onto a nitrocellulose membrane. Signals were detected by using chemiluminescence.

### Purification of His-tagged conjugates

The purification of His-tagged conjugates using Ni^2+^-NTA- agarose beads allowing the purification of proteins that are covalently conjugated to His6-SUMO, was performed as described previously^27^.

### Immunofluorescence staining

Immunofluorescence staining and confocal analysis were performed as described^28^.

### VLPs purification

Purification of VLPs was carried out as described previously^29^. Briefly, HEK-293 cells were transfected with VP40 WT or VP40K326R and 72 h after transfection, supernatants cleared from cells and cellular debris were overlaid on 20% sucrose and were ultracentrifuged at 100,000 × g for 2 h at 4 °C. The pellet was then further ultracentrifuged (100,000 × g for 2 h at 4 °C) on a sucrose step gradient (60%, 40%, and 10%). The VLPs were recovered from the interface of 10% and 40% sucrose, and analyzed by immunoblotting and electron microscopy.

### Electron Microscopy of VLPs

A drop of 5 ml of the sample was incubated on glow discharged collodion/carbon coated grids for 5 min. Excess fluid was removed with a piece of Whatman paper. Then the grid was incubated with 2% (w/v) uranyl acetate for 30 sec. After staining the grids were blotted and air dried on a filter paper in a Petri dish. The grids were examined in a JEOL JEM 1230 transmission electron microscope at 100 kV.

### Immunoelectron microscopy of VLPs

A drop of 3.5 μl of the sample was incubated on glow discharged collodion/carbon coated nickel grids for 2 min at room temperature. To unmask epitopes in the VLPs, samples were cross-linked with 0.5% paraformaldehyde for 15 min, incubated or not in 0.05% Triton X-100 for 15 min, and further cross-linked for another 15 min with 4% paraformaldehyde at 4 °C. The rest of the assay was carried out at room temperature. Unspecific binding was blocked by floating grids on a drop of TBG (30 mM Tris-HCl pH 8, 150 mM NaCl, 0.1% bovine serum albumin (BSA) and 1% gelatin) for 10 min. Grids were then incubated for 15 min with the anti-HA or anti-SUMO2 antibody (1:10 dilution) diluted in TBG and washed (4 × 2 min) in 0.1% gelatin in PBS (137 mM NaCl, 2.7 mM KCl, 10 mM Na_2_HPO_4_, 1.8 mM KH_2_PO_4_ pH 7.4). After 5 min blocking in TBG, grids were incubated for 15 min in the appropriate colloidal gold conjugated secondary antibody diluted 1:40 in TBG. Finally, grids were washed in 0.1% gelatin in PBS (5 × 2 min), milliQ water (3 × 2 min), and stained with 2% uranyl acetate (30 sec). Grids were examined in a JEOL 1230 electron microscope.

### Immunoprecipitation assay

Cells were lysed in TNN buffer (100 mM Tris-HCl, pH 8, 250 mM NaCl, 0.5% NP-40) at 4 °C, centrifuged at 15,800 × *g* for 5 min and immunoprecipitated overnight at 4 °C after addition of the specified antibody and 50 μl of 50% protein A-Sepharose CL-4B beads (GE Healthcare). Beads were then washed four times with TNN buffer and resuspended in 30 μl of SDS-PAGE loading buffer.

### Statistical analysis

For statistical analysis between the control and the different groups the Student’s t test was applied. The significance level chosen for the statistical analysis was p < 0.05.

## Additional Information

**How to cite this article**: Baz-Martínez, M. *et al.* Regulation of Ebola virus VP40 matrix protein by SUMO. *Sci. Rep.* **6**, 37258; doi: 10.1038/srep37258 (2016).

**Publisher’s note**: Springer Nature remains neutral with regard to jurisdictional claims in published maps and institutional affiliations.

## References

[b1] Baize S. *et al.* Emergence of Zaire Ebola virus disease in Guinea. N Engl J Med 371, 1418–1425 (2014). 24738640 10.1056/NEJMoa1404505

[b2] Schieffelin J. S. *et al.* Clinical illness and outcomes in patients with Ebola in Sierra Leone. N Engl J Med 371, 2092–2100 (2014). 25353969 10.1056/NEJMoa1411680 PMC4318555

[b3] Harty R. N., Brown M. E., Wang G., Huibregtse J. & Hayes F. P. A PPxY motif within the VP40 protein of Ebola virus interacts physically and functionally with a ubiquitin ligase: implications for filovirus budding. Proc Natl Acad Sci USA 97, 13871–13876 (2000). 11095724 10.1073/pnas.250277297 PMC17668

[b4] Jasenosky L. D., Neumann G., Lukashevich I. & Kawaoka Y. Ebola virus VP40-induced particle formation and association with the lipid bilayer. J Virol 75, 5205–5214 (2001). 11333902 10.1128/JVI.75.11.5205-5214.2001 PMC114926

[b5] Timmins J., Scianimanico S., Schoehn G. & Weissenhorn W. Vesicular release of ebola virus matrix protein VP40. Virology 283, 1–6 (2001). 11312656 10.1006/viro.2001.0860

[b6] Hoenen T., Jung S., Herwig A., Groseth A. & Becker S. Both matrix proteins of Ebola virus contribute to the regulation of viral genome replication and transcription. Virology 403, 56–66 (2010). 20444481 10.1016/j.virol.2010.04.002

[b7] Dessen A., Volchkov V., Dolnik O., Klenk H. D. & Weissenhorn W. Crystal structure of the matrix protein VP40 from Ebola virus. Embo J 19, 4228–4236 (2000). 10944105 10.1093/emboj/19.16.4228 PMC302032

[b8] Ruigrok R. W. *et al.* Structural characterization and membrane binding properties of the matrix protein VP40 of Ebola virus. J Mol Biol 300, 103–112 (2000). 10864502 10.1006/jmbi.2000.3822

[b9] Hoenen T. *et al.* Oligomerization of Ebola virus VP40 is essential for particle morphogenesis and regulation of viral transcription. J Virol 84, 7053–7063 (2010). 20463076 10.1128/JVI.00737-10 PMC2898221

[b10] Gomis-Ruth F. X. *et al.* The matrix protein VP40 from Ebola virus octamerizes into pore-like structures with specific RNA binding properties. Structure 11, 423–433 (2003). 12679020 10.1016/S0969-2126(03)00050-9 PMC7126486

[b11] Hochstrasser M. Origin and function of ubiquitin-like proteins. Nature 458, 422–429 (2009). 19325621 10.1038/nature07958 PMC2819001

[b12] Chang T. H. *et al.* Ebola Zaire virus blocks type I interferon production by exploiting the host SUMO modification machinery. PLoS Pathog 5, e1000493 (2009). 19557165 10.1371/journal.ppat.1000493 PMC2696038

[b13] Han Z. *et al.* ITCH E3 Ubiquitin Ligase Interacts with Ebola Virus VP40 To Regulate Budding. J Virol 90, 9163–9171 (2016). 27489272 10.1128/JVI.01078-16 PMC5044852

[b14] Okumura A., Pitha P. M. & Harty R. N. ISG15 inhibits Ebola VP40 VLP budding in an L-domain-dependent manner by blocking Nedd4 ligase activity. Proc Natl Acad Sci USA 105, 3974–3979 (2008). 18305167 10.1073/pnas.0710629105 PMC2268823

[b15] Jentsch S. & Pyrowolakis G. Ubiquitin and its kin: how close are the family ties? Trends Cell Biol 10, 335–342 (2000). 10884686 10.1016/s0962-8924(00)01785-2

[b16] Bernier-Villamor V., Sampson D. A., Matunis M. J. & Lima C. D. Structural basis for E2-mediated SUMO conjugation revealed by a complex between ubiquitin-conjugating enzyme Ubc9 and RanGAP1. Cell 108, 345–356 (2002). 11853669 10.1016/s0092-8674(02)00630-x

[b17] Rodriguez M. S., Dargemont C. & Hay R. T. SUMO-1 conjugation *in vivo* requires both a consensus modification motif and nuclear targeting. J Biol Chem 276, 12654–12659 (2001). 11124955 10.1074/jbc.M009476200

[b18] Sampson D. A., Wang M. & Matunis M. J. The small ubiquitin-like modifier-1 (SUMO-1) consensus sequence mediates Ubc9 binding and is essential for SUMO-1 modification. J Biol Chem 276, 21664–21669 (2001). 11259410 10.1074/jbc.M100006200

[b19] Muller S., Hoege C., Pyrowolakis G. & Jentsch S. SUMO, ubiquitin’s mysterious cousin. Nat Rev Mol Cell Biol 2, 202–210 (2001). 11265250 10.1038/35056591

[b20] Ulrich H. D. The fast-growing business of SUMO chains. Mol Cell 32, 301–305 (2008). 18995828 10.1016/j.molcel.2008.10.010

[b21] Boggio R. & Chiocca S. Viruses and sumoylation: recent highlights. Curr Opin Microbiol 9, 430–436 (2006). 16815735 10.1016/j.mib.2006.06.008 PMC7108358

[b22] Wimmer P., Schreiner S. & Dobner T. Human pathogens and the host cell SUMOylation system. J Virol 86, 642–654 (2012). 22072786 10.1128/JVI.06227-11 PMC3255802

[b23] Desterro J. M., Rodriguez M. S. & Hay R. T. SUMO-1 modification of IkappaBalpha inhibits NF-kappaB activation. Mol Cell 2, 233–239 (1998). 9734360 10.1016/s1097-2765(00)80133-1

[b24] Vertegaal A. C. *et al.* Distinct and overlapping sets of SUMO-1 and SUMO-2 target proteins revealed by quantitative proteomics. Mol Cell Proteomics 5, 2298–2310 (2006). 17000644 10.1074/mcp.M600212-MCP200

[b25] Campagna M. *et al.* Rotavirus viroplasm proteins interact with the cellular SUMOylation system: implications for viroplasm-like structure formation. J Virol 87, 807–817 (2013). 23115286 10.1128/JVI.01578-12 PMC3554093

[b26] Gonzalez-Santamaria J. *et al.* Regulation of the tumor suppressor PTEN by SUMO. Cell Death Dis 3, e393 (2012). 23013792 10.1038/cddis.2012.135 PMC3461367

[b27] de la Cruz-Herrera C. F. *et al.* SUMOylation regulates AKT1 activity. Oncogene 34, 1442–1450 (2015). 24704831 10.1038/onc.2014.48

[b28] de la Cruz-Herrera C. F. *et al.* Conjugation of SUMO to p85 leads to a novel mechanism of PI3K regulation. Oncogene (2015). 10.1038/onc.2015.356 26411363

[b29] Panchal R. G. *et al.* *In vivo* oligomerization and raft localization of Ebola virus protein VP40 during vesicular budding. Proc Natl Acad Sci USA 100, 15936–15941 (2003). 14673115 10.1073/pnas.2533915100 PMC307671

